# Prediction of the risk of transplant rejection based on RNA sequencing data of PBMCs before transplantation

**DOI:** 10.1038/s41598-025-09780-8

**Published:** 2025-08-04

**Authors:** Yu Gong, Yuan Wang, Kazuyoshi Takeda, Saori Hirota, Yui Maehara, Ko Okumura, Koichiro Uchida

**Affiliations:** 1 Center for Immune Therapeutics and Diagnosis, Advanced Research Institute for Health Science, Juntendo University, Tokyo, 113-0033 Japan; 2 Department of Integrated Biosciences, Graduate School of Frontier Sciences, The University of Tokyo, Kashiwa, 277-0882 Japan; 3 Laboratory of Cell Biology, Biomedical Research Core Facilities, Juntendo University Graduate School of Medicine, Tokyo, 113-0033 Japan; 4 Department of Biofunctional Microbiota, Juntendo University Graduate School of Medicine, Tokyo, 113-0033 Japan; 5 Atopy Research Center, Juntendo University Graduate School of Medicine, Tokyo, 113-0033 Japan

**Keywords:** Liver transplantation, Kidney transplantation, Rejection, Peripheral blood mononuclear cells, RNA sequencing, Transplant immunology, Computational models

## Abstract

Novel methods for detecting transplant rejection are craved, since conventional methods can detect ongoing rejection that may sometimes have already caused irreversible damage in transplanted organs. Here, we applied a transcriptomics database of recipients’ peripheral blood mononuclear cells (PBMCs) before liver or kidney transplantation on the weighted gene co-expression network and machine learning models to evaluate the risk of rejection. Gene clusters positively correlated with rejection were enriched for genes related to antiviral response and regulation/production of interleukin-1(IL-1) in liver transplantation, and genes related to innate immune responses (IL-8 and toll-like receptor signaling pathways) and T cell responses were positively correlated with rejection in kidney transplantation. Our study presents a novel approach for feature engineering based on RNA-seq data of PBMCs collected before transplantation. The features derived from this method demonstrated potential in predicting the risk of rejection and may serve as candidate predictors in future clinically applicable models.

**Supplementary Information:**

The online version contains supplementary material available at 10.1038/s41598-025-09780-8.

## Introduction

Transplant rejection is the most serious complication after organ transplantation^1,2^. When not detected or treated in a timely manner before the serious injury, rejecting reactions would lead to poor prognosis, failure of transplanted organ functions and an increased risk of patient death^3^. Rejecting reactions have been monitored by blood tests, function analysis of transplanted organ, and/or histological examination of the biopsy in clinical^4–6^. These methods have their own advantage and can complement each other to improve diagnostic accuracy, however, all these methods have a certain degree of delay to detect rejecting reactions. Particularly in the transplantation of organs that cannot regenerate, such as kidneys, prediction of rejection is required to avoid irreversible damage leading to transplant failure^7,8^.

Recently, rapid advances in DNA and/or RNA-seq technology became powerful tools for more accurate diagnosis in transplant medicine, and several genomic biomarkers have been reported to detect graft dysfunction^9–11^. Polymorphisms including single nucleotide polymorphisms (SNPs) in some genes have been reported to correlate with rejecting reactions^12,13^. Moreover, the rapid development of machine learning, which includes many excellent regression models and classifiers, has been contributing to developing novel methods to predict the patient prognosis in some diseases^14,15^. Thus, we are now able to analyze a large number of gene expression data from patient samples based on rationally related pathways and might be able to predict the risk of rejection more accurately using these technologies.

Here, we divided the RNA-seq data of patients’ PBMCs before transplantation into several modules related to rejecting reactions by establishing a weighted co-expression network and calculated the scores of each module. The scores of the modules were used as features to train machine learning models. The utility of this method was validated in two datasets, one for liver transplantation and the other for kidney transplantation. The features demonstrated a certain degree of predictive ability for rejection risk in both datasets.

## Materials and methods

### Data collection and processing of RNA-seq datasets

We collected RNA sequencing (RNA-seq) data from 75 blood samples of pre-liver transplantation, which was available from the Gene Expression Omnibus (GEO accession number: GSE200340)^16^. The expression levels were normalized using the ‘NormalizeData’ function in the Seurat package^17^. In addition, we obtained a dataset consisting of RNA-seq data from 155 kidney transplant patients before transplantation, also from the Gene Expression Omnibus (GEO accession number: GSE112927)^14^. Definitions of rejection were based on standard clinical practice and relevant guidelines for each type of organ transplantation. Briefly, in the pediatric liver transplant dataset, predicted rejection referred to early acute cellular rejection (ACR) occurring within 3 months post-transplant. In the adult kidney transplant cohort, predicted rejection was defined as biopsy-proven ACR occurring within 6 months post-transplant, according to the Banff 2013 classification.

### Weighted gene co-expression analysis

A co-expression network was constructed using the weighted gene co-expression network analysis (WGCNA) package in R^18^. When performing WGCNA on GSE200340, which represents gene expression levels in fragments per kilobase of exon model per million mapped fragments (FPKM), genes with low expression (FPKM sum less than 50) and genes expressed only in a small number of cells (expressed in less than 2% of cells) were excluded. The soft-threshold power used to create the pairwise distance matrix was defined as the lowest power for which the scale-free topology fit index reaches 0.82. The package “blockwiseModules” was used to automatically form unsigned networks and detect modules. The “cor” function was used to calculate the correlation coefficient between the module and the phenotype (rejectors “R” or non-rejectors “NR”), as well as the geneTraitSignificance (GS) and GS p-value of genes within each module. We applied the false discovery rate (FDR) method to adjust the GS p-value.

### Protein-protein interaction networks construction and functional enrichment analysis

The protein-protein interaction network of genes contained in the modules was analyzed using the online tool STRING. Clustering analysis of the protein-protein interaction network was performed using MCL clustering with an inflation parameter of 3^19^. Enrichment analysis was then performed for the genes contained in each cluster in R.

### Machine learning model based on WGCNA

To reduce noise, we set thresholds for GS and GS FDR to limit the number of genes used for module factor (MF) calculation. The threshold selection was based on two principles:

1. Ensuring that each module contains a sufficient number of genes;
2. Ensuring that each module’s MF retains the ability to distinguish between R and NR samples.

A GS FDR threshold of < 0.4 was applied, under which the selected modules in both the liver and kidney transplant datasets retained a sufficient number of genes (Fig. S1a, b). As increasing the GS threshold reduced the discriminatory power of certain modules between R and NR samples, evidenced by increased adjusted P-values starting from GS > 0.1 (Fig. S1c, d), genes within each module were selected using the thresholds GS > 0.1 and FDR < 0.4.

In module selection, we required that each MF be capable of distinguishing between R and NR samples. We also ensured that the number of modules positively associated with R was comparable to those associated with NR. To minimize the risk of overfitting from irrelevant features, the number of features was set to the minimum value that could significantly improve the model’s predictive power. For liver transplantation, the optimal number of features was 5, and for kidney transplantation, it was 6 (Fig. S1e, f).

The following formula was used to calculate MF:

1 $$MF = \mathop \sum \limits_{{i = 1}}^{n} GS_{i} EX_{i}$$

where *MF* stands for module factor, which was used as a feature for training the machine learning model. *GS*_*i*_ is the correlation between *gene*_*i*_ and trait calculated in WGCNA. *EX*_*i*_ is the expression values of the *gene*_*i*_. The number of MFs depends on the number of modules selected in WGCNA. Another summarization strategy was prepared for visualization:

2 $$MF_{R} = \mathop \sum \limits_{{j = 1}}^{n} MF_{j}$$

3 $$MF_{{NR}} = \mathop \sum \limits_{{k = 1}}^{n} MF_{k}$$

where *MF*_*j*_ represents the MF of module that is positively correlated with R. *MF*_*k*_ represents the MF of module that is positively correlated with NR. In order to prevent the influence of modules containing highly expressed genes or a large number of genes, the MF values were normalized by the function of min_max_norm.

The dataset was split into training and test sets in a 4:1 ratio using stratified random sampling. The training set was used to train models including support vector machine (SVM), k-nearest neighbor (KNN), Logistic regression, perceptron and decision tree^20–23^. Ten-fold cross-validation was performed on the models, and ultimately the logistic regression model with the highest accuracy was selected to predict on the test set. Considering the small size of the dataset, the randomness of the data division may affect the prediction results. Therefore, the training set and test set were randomly divided 50 times, and the models were independently trained and predicted on the training and test set. Additionally, we applied L2 regularization to further reduce the risk of overfitting.

### Statistics

Data analysis, statistical test and visualization were conducted in R (version 4.1.3) and GraphPad Prism (v7.0). Statistical analysis was performed by using multiple unpaired t-tests to compare the MFs of R with those of NR. * *p* < 0.05, ** *p* < 0.01, *** *p* < 0.001, **** *p* < 0.0001.

## Results

### Weighted co-expression network based rejection prediction strategy

**Fig. 1:**
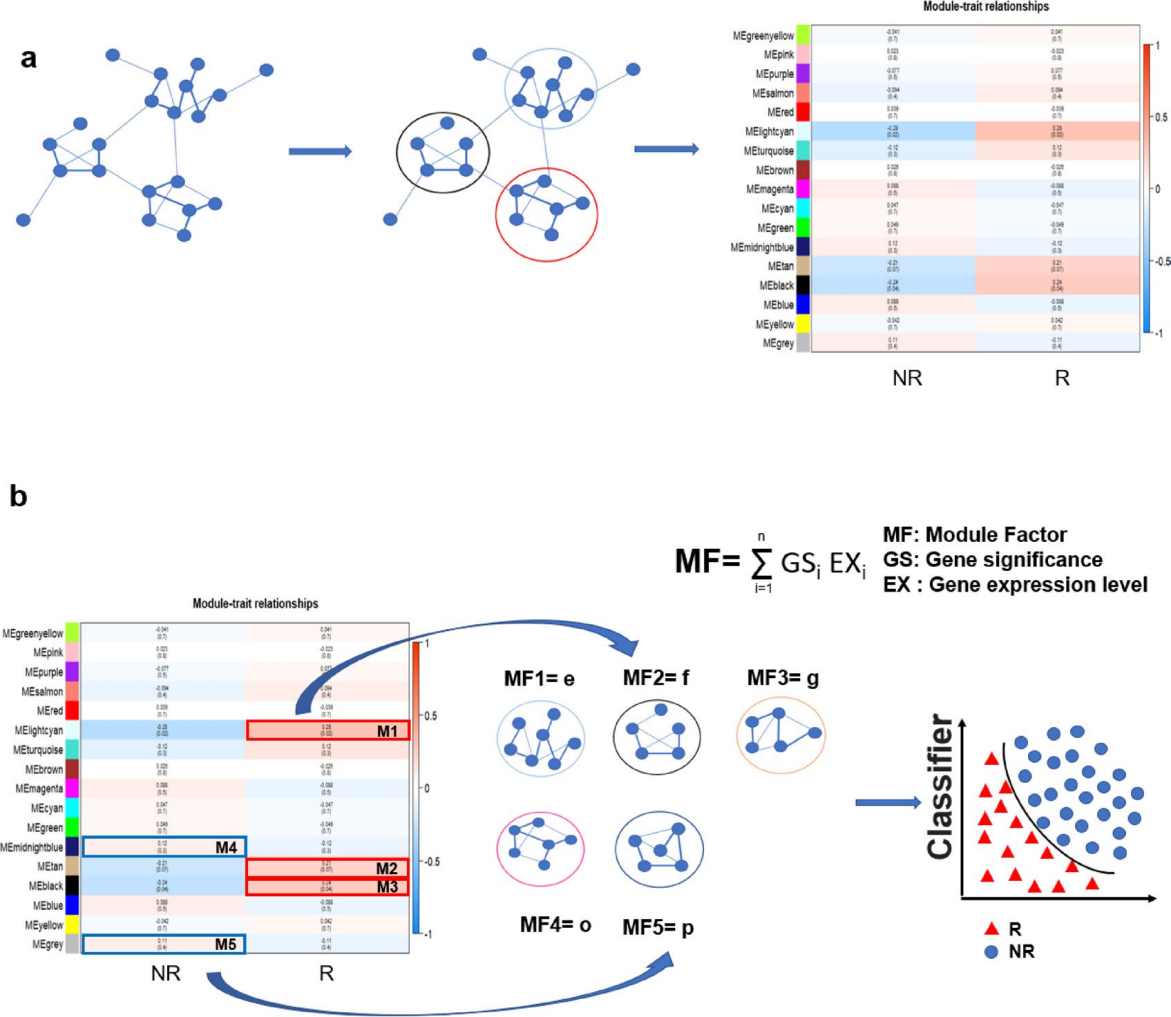
Weighted co-expression network based rejection prediction strategy. (**a**) Identifying modules in weighted co-expression networks that are related to traits (NR and R). (**b**) Using gene expression levels and gene significance in modules to perform feature engineering, and using the computed features to train and predict on machine learning models.

To predict rejection reactions using RNA-seq data, it is necessary to convert tens of thousands of gene expression data into the features related to rejecting reactions. One approach is to find differentially expressed genes (DEGs) between rejectors and non-rejectors and use DEGs as separate features for prediction^24^. Even after a certain screening of DEGs, dozens or even hundreds of DEGs may remain. In this case, a large number of features often leads to a very complex model, which would result in serious overfitting during model training for prediction, especially when the amount of training data is limited such as transplant rejection^25^. To avoid this problem, we developed a new conceptual paradigm in that we first construct a scale-free network and divide the network into modules. Then, we correlated the traits (R or NR) to gene modules (Fig. 1a). Finally, the expression levels of each module are calculated and used as features for training machine learning models (Fig. 1b). Compared to training machine learning models with DEGs, this weighted co-expression network could reduce overfitting and minimize the impact of noise on machine learning models by reducing dimensionality. Moreover, calculating the correlation between each gene and phenotype and adjusting the weight of each gene could retain biological significance.

### Peripheral blood transcriptomic signatures associated with liver rejection

**Fig. 2:**
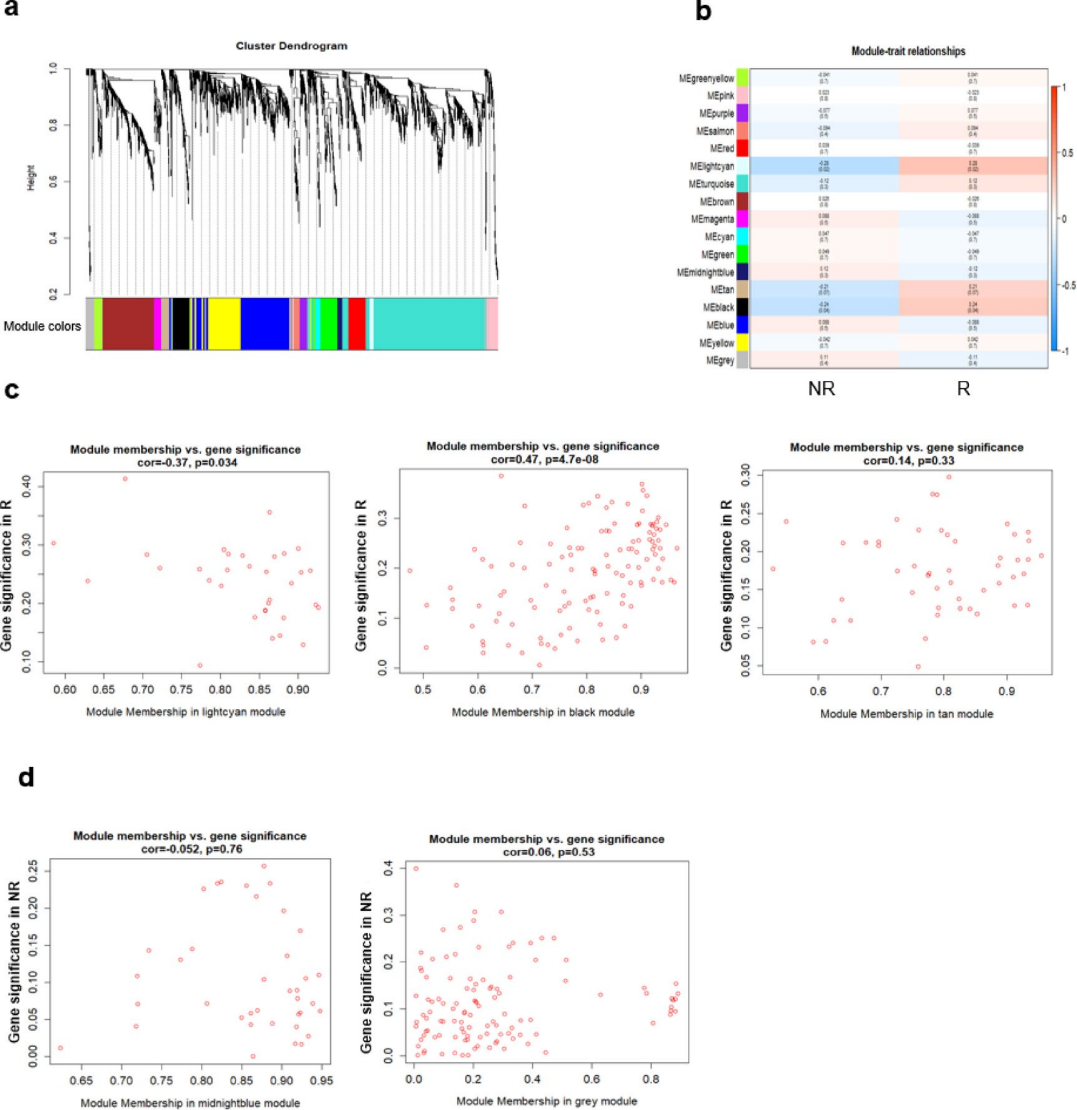
Identified and analyzed trait-related modules of LT in co-expression network. (**a**) WGCNA in LT patient-derived PBMCs, showing a hierarchical clustering tree of co-expression modules. Each module corresponds to a branch, which is labeled by a distinct color shown underneath. (**b**) WGCNA identifies 17 modules with highly correlated gene expression patterns in R and NR. Correlations between each module and R or NR are indicated by the intensity of red or blue color, respectively. p value for each module is shown in brackets. (**c**) The module membership and gene significance of genes within modules positively correlated with R. Each dot represents a gene within the corresponding module. (**d**) The module membership and gene significance of genes within modules positively correlated with NR. Each dot represents a gene within the corresponding module.

Here we present an example of using this method for prediction. We applied this method to a publicly available sequencing dataset from PBMCs before liver transplantation (LT) (GEO accession number: GSE200340). Children experiencing rejection within 90 days after LT were termed R. NR did not experience rejection during this period. First, the samples were clustered and no obvious outlier was found (Fig. S2a). A soft threshold of 10 was chosen to construct a gene co-expression network (Fig. S2b). Then, 17 co-expression modules were identified and correlated with the traits (R or NR) (Fig. 2a, b). Particularly, we identified three co-expression modules containing 205 genes positively correlated with R. (*n* = 33 for light cyan; *n* = 122 for black and *n* = 50 for tan expression modules) and two co-expression modules containing 152 genes positively correlated with NR (*n* = 38 for midnight blue and *n* = 114 for grey expression modules) (Fig. 2c, d). Therefore, by constructing a weighted gene co-expression network and linking it to the traits, we identified genes associated with R or NR and obtained the GS which was defined as the correlation between gene expression and the traits.

**Fig. 3:**
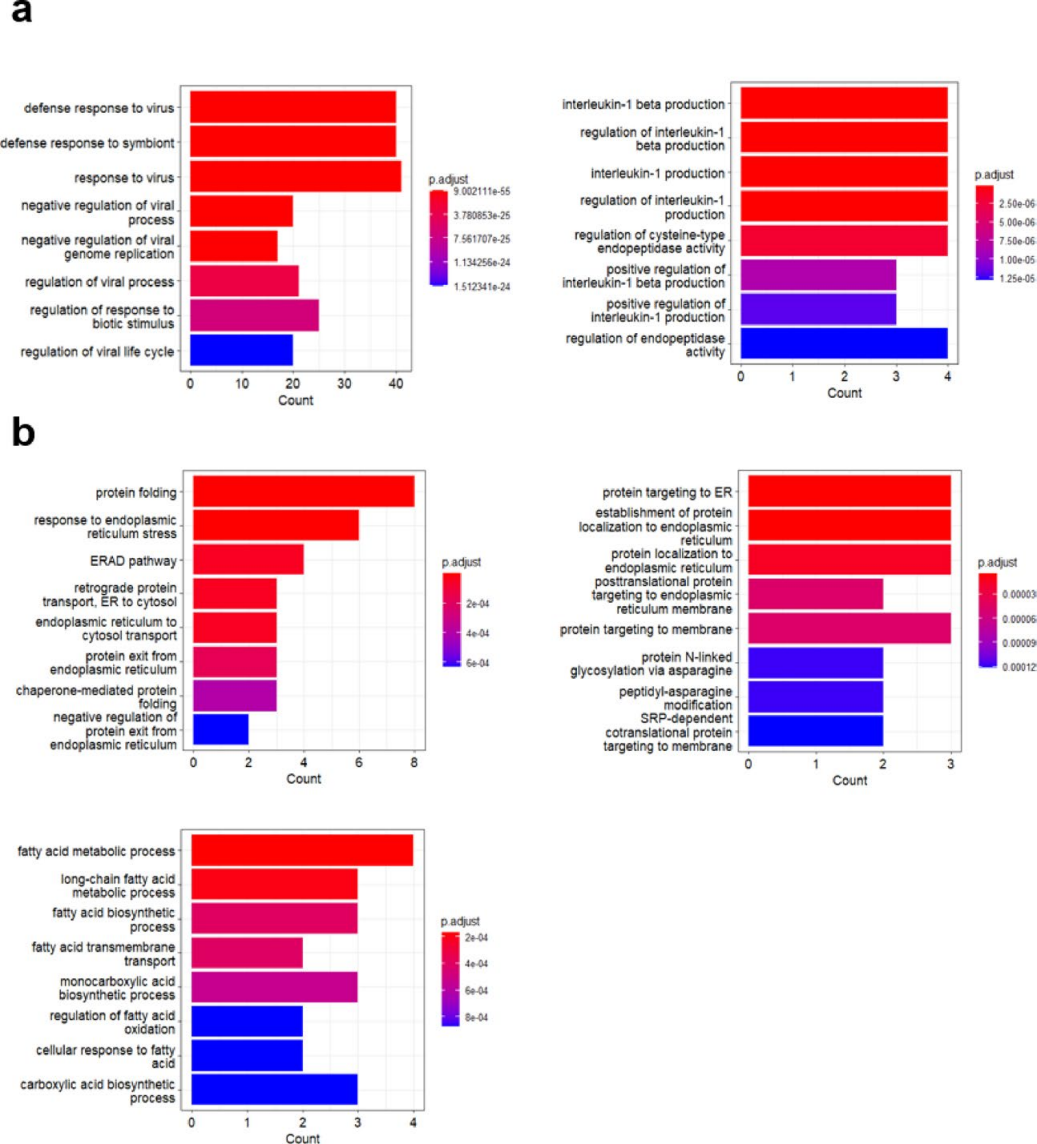
Analysis of gene modules of LT related to R or NR. (**a**) GO term analysis was performed on transcripts that were enriched in modules related to R. The y-axis represents the GO terms, the x-axis represents the number of assigned genes. (**b**) GO term analysis was performed on transcripts that were enriched in modules related to NR. The y-axis represents the GO terms, the x-axis represents the number of assigned genes.

### Identifying network modules related to LT rejectors or non-rejectors

We then conducted an analysis of the modules related to R and NR. First, we clustered the genes in each module, and then conducted gene ontology (GO) analysis on the major clusters with more than 5 genes. In the black module, which is positively correlated with R, we identified two clusters that were enriched for genes related to antiviral response and regulation/production of interleukin-1 (IL-1) (Figs. 3a, S3a). However, we did not find any clusters with more than 5 genes in the light cyan and tan modules, although there were small clusters (Fig. S3c). This may be due to the fewer number of genes in these modules or less research on the genes in these modules. On the other hand, in the midnight blue and grey modules that are positively correlated with NR, we found three clusters that were enriched for genes related to endoplasmic reticulum activity and fatty acid metabolism (Figs. 3b, S3b). To reduce noise, we selected genes that were highly correlated with R or NR (gene significance > 0.1, FDR < 0.5), and we identified the modules and genes associated with R and NR, and these selected genes will be used to calculate the features for training the machine learning model.

### Feature engineering and prediction of liver transplant rejection based on network modules

Although using DEGs as features to train the machine learning model does not require complex feature engineering, a large number of independent features can introduce noise and cause the model to overfit. In such cases, feature engineering can be used to add features that better describe the data characteristics and improve the quality of the machine learning results.

**Fig. 4:**
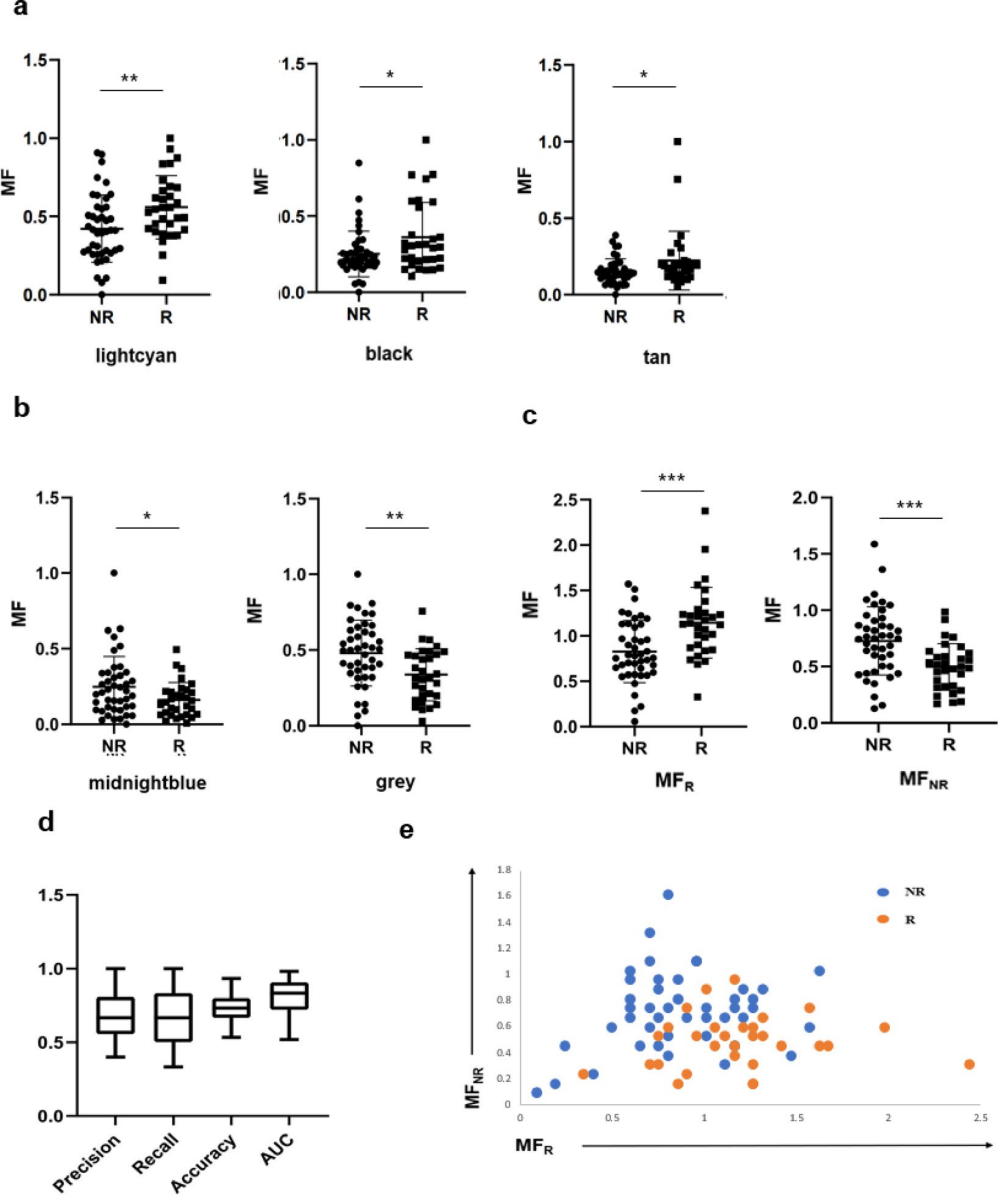
The MF calculated based on gene modules of LT has discriminative power for R and NR. (**a**) The MF of each module that is positively correlated with R. (**b**) The MF of each module that is positively correlated with NR. (**c**) The 2 new MF generated by summing the MFs of the corresponding modules in (**a**) and (**b**). (**d**) Using precision, recall, accuracy and AUC to evaluate the predictive power of the MF in (**a**) and (**b**). (**e**) Using the MF in (**c**) to discriminate between R and NR subjects.

We used gene expression values and GS obtained in the WGCNA process to calculate the MF for each module, and used the MFs as the features for training and prediction. This reduces the dimensions of the data to the same number of selected modules, greatly reducing the number of features. We created these features based on the three modules that are positively correlated with R (Fig. 4a), and the two modules that are positively correlated with NR (Fig. 4b). These MFs are significantly higher in R or NR respectively. Since the number of features exceeds three, it is not possible to visualize the position of each point in a two- or three-dimensional coordinate system. In order to more intuitively determine the level of rejection risk for a patient, the MFs can be summed to create MF_R_ and MF_NR_, which are the merged features that are positively correlated with R and NR, respectively. These two merged features become more significant in R and NR (Fig. 4c). To evaluate the predictive ability of our approach, we performed k-fold cross-validation on several binary classifiers and selected the logistic regression to make predictions on the data in the database. When there is a large amount of data, it is usually common to randomly split the data into training and test sets, using the training set to train the model and the test set as a final check on the model’s prediction ability. However, in the case of a small amount of data, the randomly obtained test set may not represent the distribution of the entire data, resulting in fluctuations in the accuracy of the binary classification model when predicting different test sets. To avoid this possibility, we randomly divided the data to training dataset and test dataset and performed training and prediction ten times. Then the average of these ten independent predictions was calculated and evaluated the model’s prediction ability.

We evaluated model performance using AUC and carried out predictions on the test set. These prediction results were evaluated by precision, recall, accuracy and AUC (Fig. 4d). The visualization of this dataset using merged feature values showed that R is concentrated in the lower right quadrant (Fig. 4e). The risk of rejection could be referred to both the prediction results and the patient’s position on the visualization graph.

In the source paper for this dataset, the median AUC is less than 0.7, while our method yields an median AUC of 0.833^16^. This suggests that our method could contribute to improved prediction of rejection reactions. Our method also provides clearer data visualization, with R concentrated in the lower right and NR concentrated in the higher left. There won’t be a situation where R and NR are clustered in different positions across different datasets^16^.

### Peripheral blood transcriptomic signatures associated with kidney rejection

**Fig. 5:**
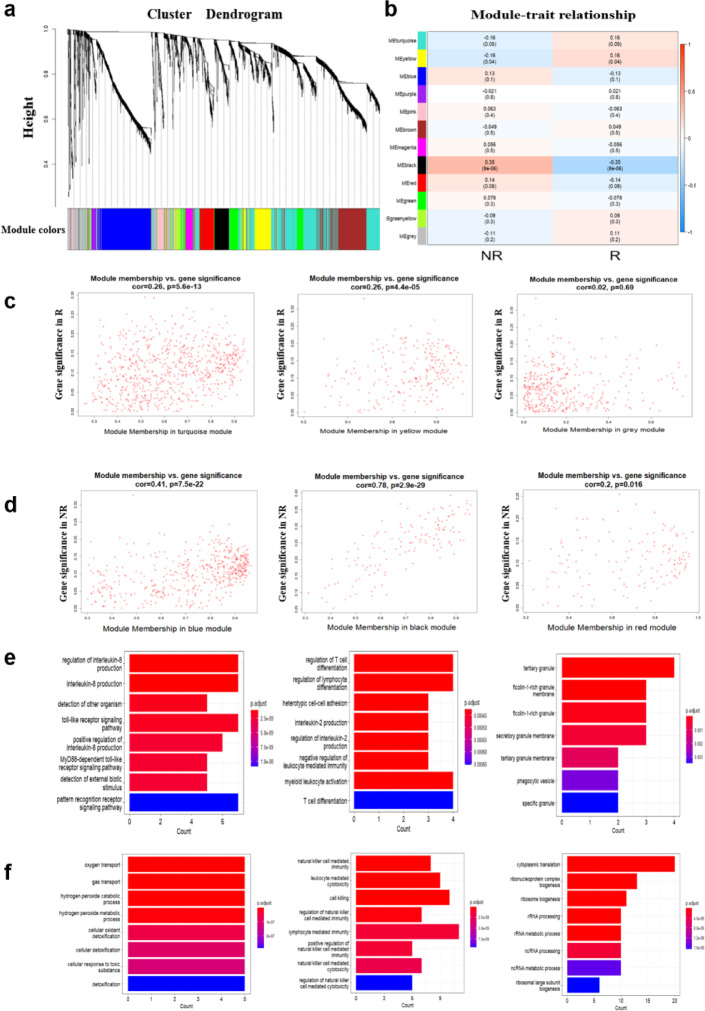
Identified and analyzed trait-related modules of KT in co-expression network. (**a**) WGCNA in KT patient-derived PBMCs, showing a hierarchical clustering tree of co-expression modules. Each module corresponds to a branch, which is labeled by a distinct color shown underneath. (**b**) WGCNA identifies 12 modules with highly correlated gene expression patterns in R and NR. Correlations between each module and R or NR are indicated by the intensity of red or blue color, respectively. p value for each module is shown in brackets. (**c**) The module membership and gene significance of genes within modules positively correlated with R. Each dot represents a gene within the corresponding module. (**d**) The module membership and gene significance of genes within module es positively correlated with NR. Each dot represents a gene within the corresponding module. (**e**) GO term analysis was performed on transcripts that were enriched in modules related to R. The y-axis represents the GO terms, the x-axis represents the number of assigned genes. (**f**) GO term analysis was performed on transcripts that were enriched in modules related to NR. The y-axis represents the GO terms, the x-axis represents the number of assigned genes.

**Fig. 6:**
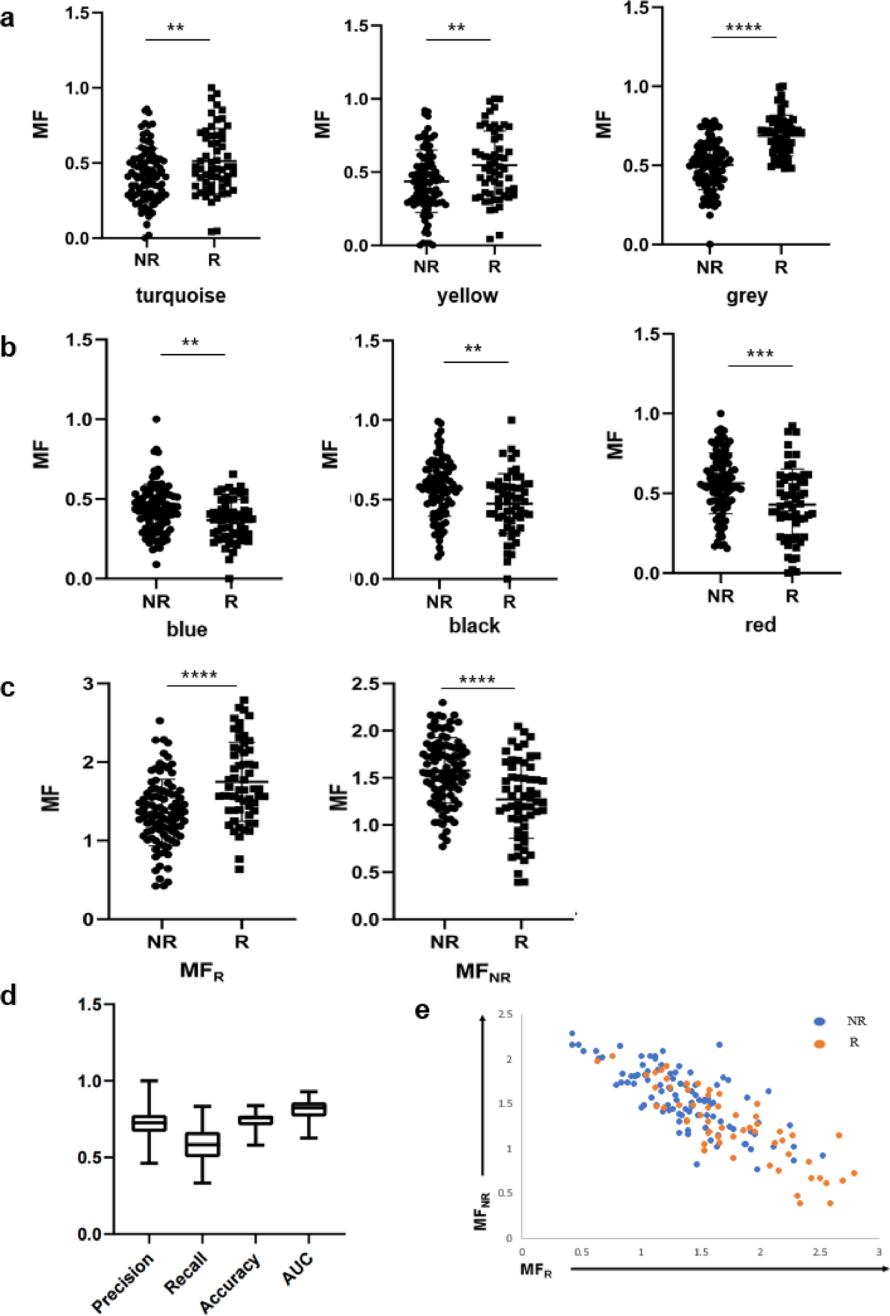
The MF calculated based on gene modules of KT has discriminative power for R and NR. (**a**) The MF of each module that is positively correlated with R. (**b**) The MF of each module that is positively correlated with NR. (**c**) The 2 new MF generated by summing the MFs of the corresponding modules in (**a**) and (**b**). (**d**) Using precision, recall, accuracy and AUC to evaluate the predictive power of the MF in (**a**) and (**b**). (**e**) Using the MF in (**c**) to discriminate between R and NR subjects.

We finally applied the same prediction method on a dataset consisting of RNA-seq data of 155 kidney transplant patients before transplantation obtained from the Gene Expression Omnibus (GEO accession number: GSE112927). Gene co-expression network was constructed (Fig. S4). Similarly, the identified 12 modules are associated with phenotypes (R or NR) (Fig. 5a, b). Here, we selected three modules positively correlated with R (module turquoise, yellow and grey) and three modules positively correlated with NR (module blue, black and red) (Fig. 5c, d). As with the modules in LT, the modules in kidney transplantation (KT) kept higher biological significance. Genes concerning innate immune responses (IL-8 and toll-like receptor signaling pathways) and T cell responses were shown to correlate with R, while genes related to gases transports, oxidant and cell cytotoxicity including natural killer cell (NK cell) activity were demonstrated to correlate with NR (Figs. 5e, f, S5). Subsequently, the MFs of these six modules were calculated, and each MF showed a certain degree of discrimination (Fig. 6a, b). To visualize the magnitude of rejection risk, the six MFs were combined into MF_NR_ and MF_R_ (Fig. 6c). We evaluated model performance using AUC and carried out predictions on the test set. These prediction results were evaluated by precision, recall, accuracy and AUC (Fig. 6d). The visualization of the rejection risk in the KT dataset also showed that the samples were more tightly clustered (Fig. 6e).

Our method achieved a median AUC of 0.825 for predicting R, and the median AUCs of 0.8 and 0.74 reported in the source paper for the discovery set and validation set, respectively.

The results demonstrated comparable performance to the previously reported approach, with a slight trend toward improved predictive accuracy.

## Discussion

Prediction and/or early detection of rejecting reactions are needed for successful transplant medicine. In this context, liquid biopsy is attractive, since samples can be obtained quickly, easily, repeatable and rather safely. In liver transplantation, rejecting reactions can be monitored by serum AST and ALT levels, and the transplanted liver could be maintained due to its regenerative ability when immune suppressive drugs are increased^26^. However, the majority of used biochemical indicators are derived from injured organs, thus the potential risk of rejection cannot be generally predicted before the starting of rejecting reaction. In kidney transplantation, transplanted kidneys sometimes fail due to their poor regenerating activity, even though creatinine has been used to monitor rejection reactions^27^. Thus, prediction of the potential risk of rejection is craved particularly before transplantation to improve the prognosis in transplant medicine.

In this study, to propose a novel method to predict the risk of rejection based on the RNA-seq results of blood samples obtained from recipients at the transplantation surgery, we constructed a weighted gene co-expression network to assort the sequencing data into several modules related to rejection and calculated scores for each module. Then, to reduce the impact of individual genes that would cause noise observed in the traditional methods using DEGs^24^, we used this as a feature to train machine learning models and decreased the number of features to reduce overfitting even if the sample number is limited. We used two datasets, datasets of blood samples collected before LT or KT to predict the risk of rejection, and showed that this method can retain the transcriptomic data differences between R and NR and make relatively accurate predictions.

This analysis method that we used is based on gene networks and thus would have a higher biological significance compared with individual genes^28–30^. In the analysis of the LT dataset, patients with a higher level of defense response to virus and IL-1 pathway were demonstrated as high risk of rejection. Type-I Interferon-induced proteins such as IFIT1, IFIT3, IFIT5, IFI6, IFI44, IFITM1, and IFITM3 are included in the related genes as a higher level of defense response to virus, suggesting that pre-existing higher level of type-I Interferon would relate with rejecting immune responses, although it was reported that the levels of INF-γ in the serum may not predict rejection^31–33^. IL-1 is also a pro-inflammatory cytokine associated with early immune responses, and IL-1 receptor antagonists have been shown to suppress rejection reactions^34–37^. Of note, although all the pathways positively correlated with R or NR are not identical between the LT and KT datasets, some pathways positively correlated with R would be functionally similar. In the analysis of the KT dataset, patients with a higher level of Toll-like receptor signaling pathways were demonstrated as high risk of rejection. Toll-like receptor signaling pathways are well known to play important roles in the immune responses against viruses and to induce the production of pro-inflammatory cytokines such as type-I IFNs, IL-1, and IL-6^38–41^. Thus, pre-existing of such pro-inflammatory cytokines would be closely related to the induction of increased immune responses against transplanted, even if the transplanted organ (liver and kidney) and the recipient age (children and adults) are different.

Patients with a higher level of fatty acid metabolism, protein folding and endoplasmic reticulum activity would be expected to be NR in LT. It was reported that proteins related to the endoplasmic reticulum stress response have higher expression in blood samples of patients experiencing R compared to those NR, although the specific mechanism is still unclear^42,43^. Here, we demonstrated that the higher expression level of CPT1a, an enzyme contributing to fatty acid metabolism, was related to NR (Fig. S3b). It has been shown that the CPT1a-related pathway is enhanced in tolerogenic DCs, and inhibition of CPT1a affects the function of tolerogenic DCs to differentiate naïve CD4 T cells into Th1 rather than Treg cells^44^. On the other hand, other studies have shown that the CPT1a inhibitor etomoxir can suppress the differentiation of monocytes into dendritic cells and macrophages, thereby reducing rejection^45^. Thus, further experiments to reveal the cell population with high expression of Cpt1a would be required to reveal the roles of fatty acid metabolism for rejecting reactions against the liver. In kidney transplantation, cell-mediated cytotoxicity-related genes, including regulation of NK cell activity, were upregulated correlating with NR. Notably, not only cellular cytotoxicity-related genes, including PRF1 and GAMA, but also genes for inhibitory and/or exhausted markers, such as PDCD1, LAG3 and TIGIT, were upregulated. Thus, this gene expression profile might suggest that cytotoxic cells, including NK cells, are exhausted and relatively dysfunctional, and that would be beneficial for NR. We should understand the limitation of the analysis using RNA-seq and would need to carefully consider the function of individual genes including gene networks (co-expression modules).

We acknowledge that a limitation of our study is the lack of adjustment for clinical confounders (e.g., age, HLA mismatch, immunosuppression), which can influence both gene expression and rejection outcomes^46^. However, because these factors influence a broad range of biological responses, their downstream effects are likely to be at least partially embedded within the transcriptomic profiles. In this sense, gene expression data might capture the influence of unmeasured clinical and biological variables, allowing for indirect inference of rejection risk. As previously discussed, studies have shown that pre-transplant pro-inflammatory cytokine levels are associated with rejection risk, regardless of recipient age (children vs. adults) or transplanted organ (liver vs. kidney). This supports the idea that gene expression can reflect certain clinical confounders. Nonetheless, the primary innovation of our study is the development of the MF metric, which represents a novel approach to feature engineering of gene expression data. In future work, we plan to incorporate clinical confounders alongside MF as input features in machine learning models to improve predictive performance by combining molecular and clinical information.

Another limitation is the relatively small sample size and the lack of independent validation. Although we employed cross-validation, regularization, and repeated random splitting of the dataset into training and test sets, the risk of overfitting remains a concern when working with limited data. We believe that an independent validation cohort is essential to determine whether overfitting occurs when using MF to predict rejection. In future studies, we aim to use our data as an independent validation set for other cohorts to assess the risk of overfitting, and to optimize our approach by integrating clinical confounders and additional features identified in other studies.

Currently, our study primarily demonstrates the potential of pre-transplant blood RNA sequencing for predicting post-transplant rejection risk and introduces a novel feature engineering strategy that could contribute to future machine learning models for clinical application.

## Electronic supplementary material

Below is the link to the electronic supplementary material.

Supplementary Material 1

## Data Availability

This study does not involve the generation of new sequencing data. Instead, it is based on the reanalysis of previously published datasets, which are available in the Gene Expression Omnibus under accession numbers GSE200340 and GSE112927. The code and additional information are available from the corresponding author upon reasonable request.

## Abbreviations

DEGs: Differentially expressed genes
FDR: False discovery rate
FPKM: Fragments per kilobase of exon model per million mapped fragments
GaussianNB: Gaussian Naive Bayes
GO: Gene ontology
GS: GeneTraitSignificance
IL-1: Interleukin-1
KT: Kidney transplantation
KNN: k-nearest neighbor
LT: Liver transplantation
MF: Module factor
NR: Non-rejectors
PBMCs: Peripheral blood mononuclear cells
R: Rejectors
RNA-seq: RNA sequencing
SNPs: Single nucleotide polymorphisms
SVM: Support vector machine
WGCNA: Weighted gene co-expression network analysis
