# Supplementary material for: Prediction of the risk of transplant rejection based on RNA sequencing data of PBMCs before transplantation
Source: Sci Rep. 2025 Aug 4;15:28391. doi: 10.1038/s41598-025-09780-8 (PMC12322293; doi:10.1038/s41598-025-09780-8)
Supplement: Supplementary file 1 — Supplementary Material 1 [file 41598_2025_9780_MOESM1_ESM.pdf]

Figure S1

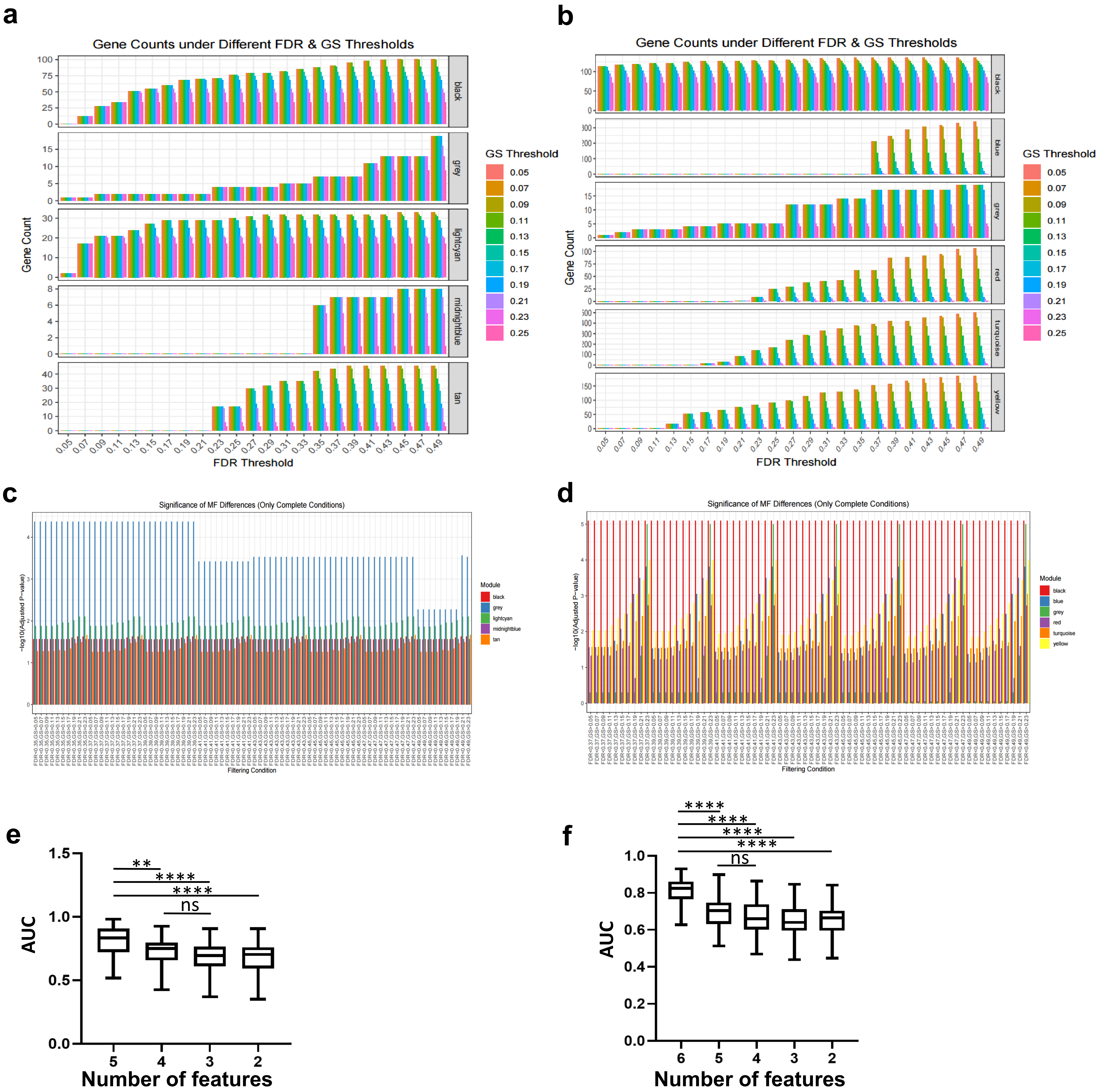

FIGURE S1 Determination of thresholds for GS and FDR. (a) The number of genes within modules under different FDR and GS thresholds in the liver transplant dataset. (b) The number of genes within modules under different FDR and GS thresholds in the kidney transplant dataset. (c) The discriminatory power of certain modules for distinguishing R and NR samples in the liver transplant dataset . (d) The discriminatory power of certain modules for distinguishing R and NR samples in the kidney transplant dataset . (e) The predictive performance of the models with different numbers of features in the liver transplant dataset. (f) The predictive performance of the models with different numbers of features in the kidney transplant dataset.

Figure S2

a b

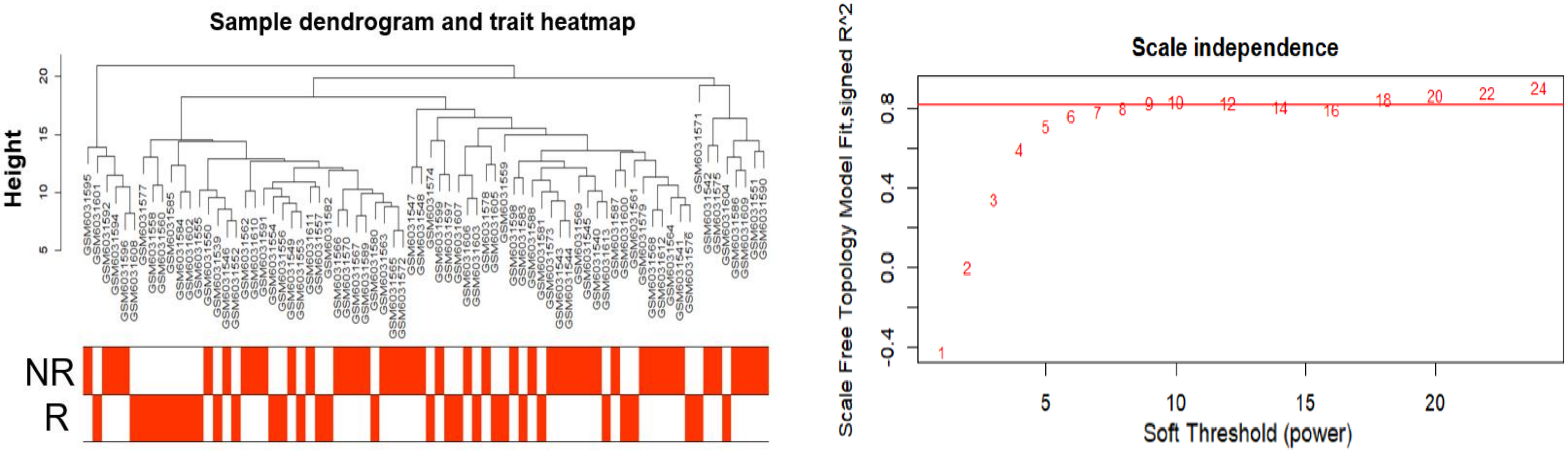

FIGURE S2 Gene co-expression network construction with LT samples. (a) Clustering analysis of PBMCs samples and their traits. The clinical traits; R and NR are shown at the bottom. (b) Determination the soft threshold for building co-expression network. The soft-threshold power used to create the pairwise distance matrix was defined as the lowest power for which the scale-free topology fit index reaches 0.82.

Figure S3

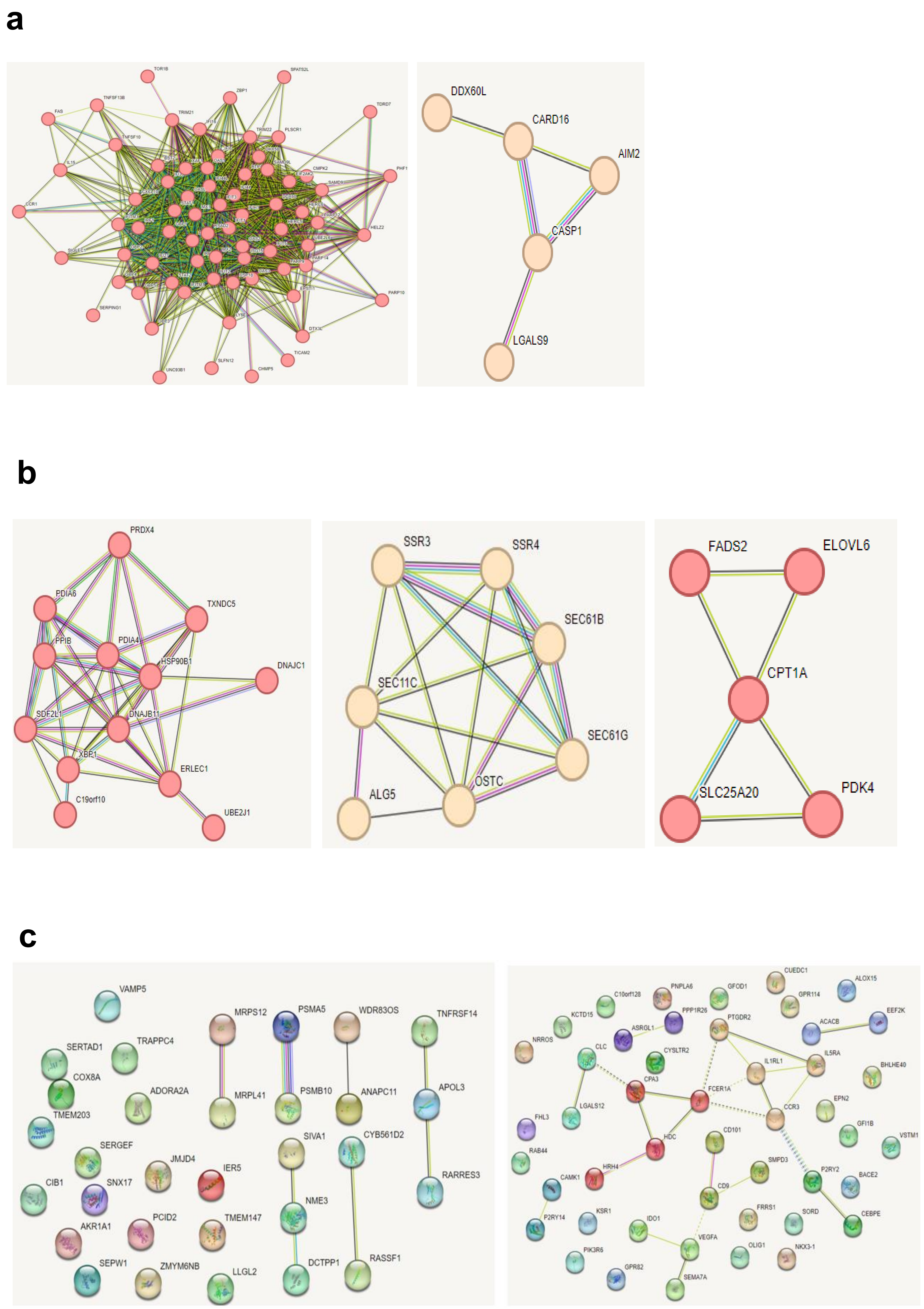

FIGURE S3 Gene network analysis of modules in LT related to R or NR. (a) The visualization of the gene network in the black modules that are positively correlated with R. (b) The visualization of the gene network in the midnightblue and grey modules that are positively correlated with NR. (c) The visualization of the gene network in the Lightcyan and Tan modules that are positively correlated with NR.

Figure S4

a **b**

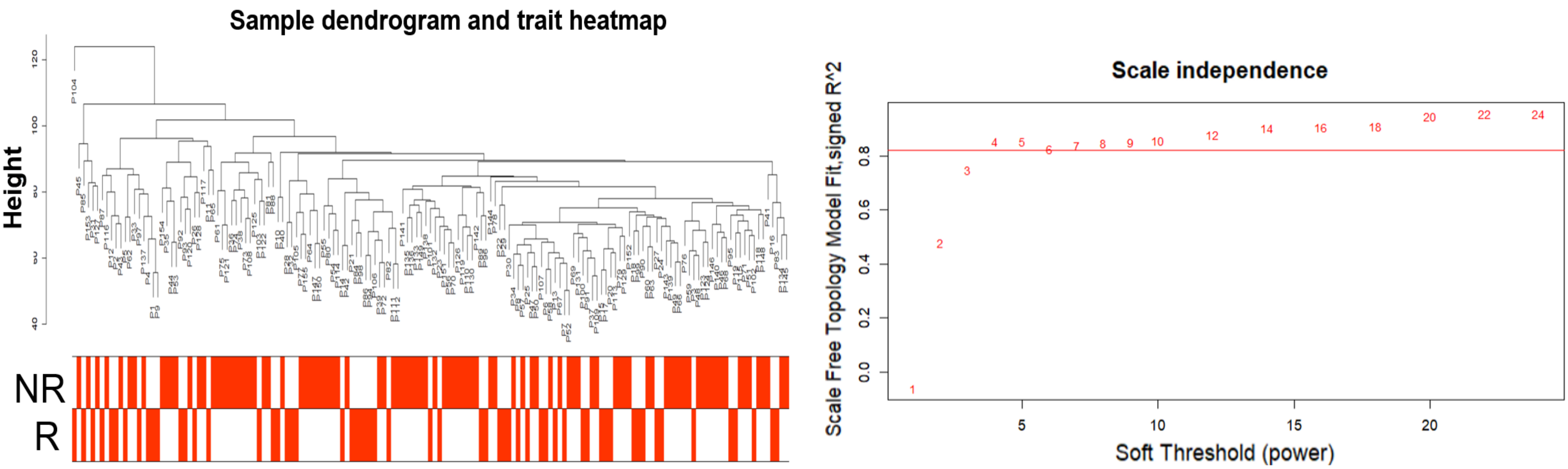

FIGURE S4 Gene co-expression network construction with KT samples. (a) Clustering analysis of PBMCs samples and their traits. The clinical traits; R and NR are shown at the bottom. (b) Determination the soft threshold for building co-expression network. The soft-threshold power used to create the pairwise distance matrix was defined as the lowest power for which the scale-free topology fit index reaches 0.82.

Figure S5

a

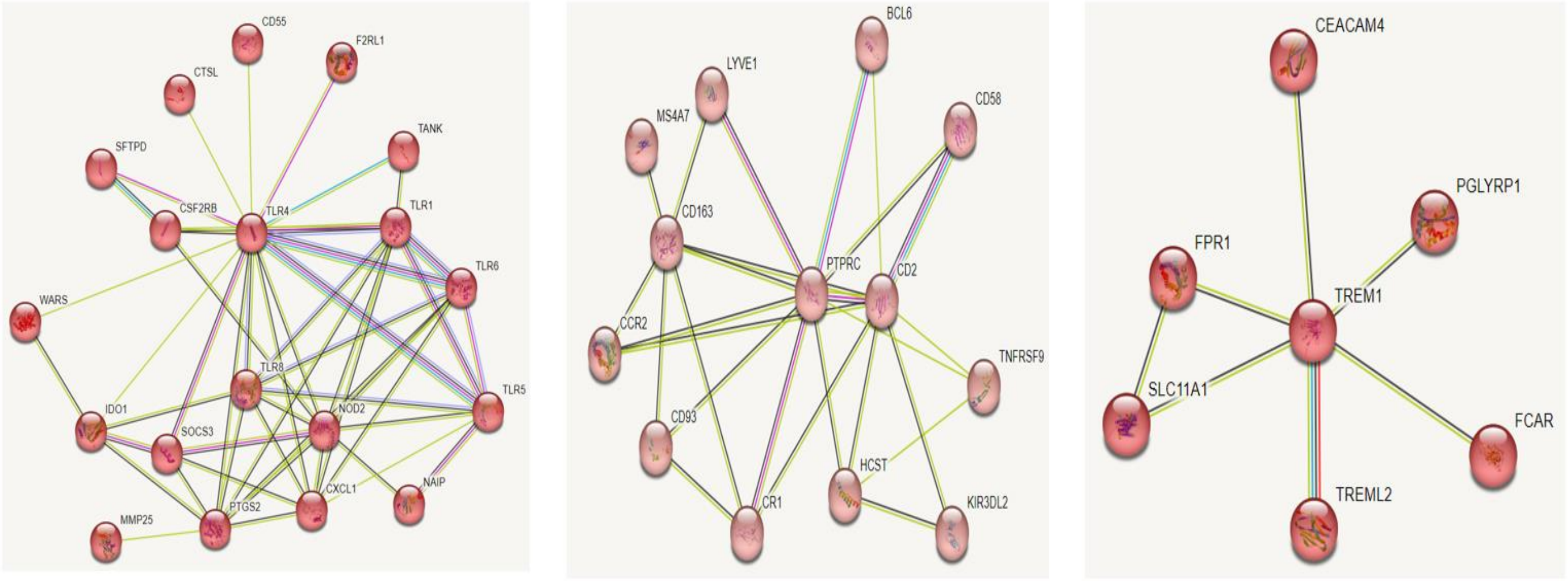

b

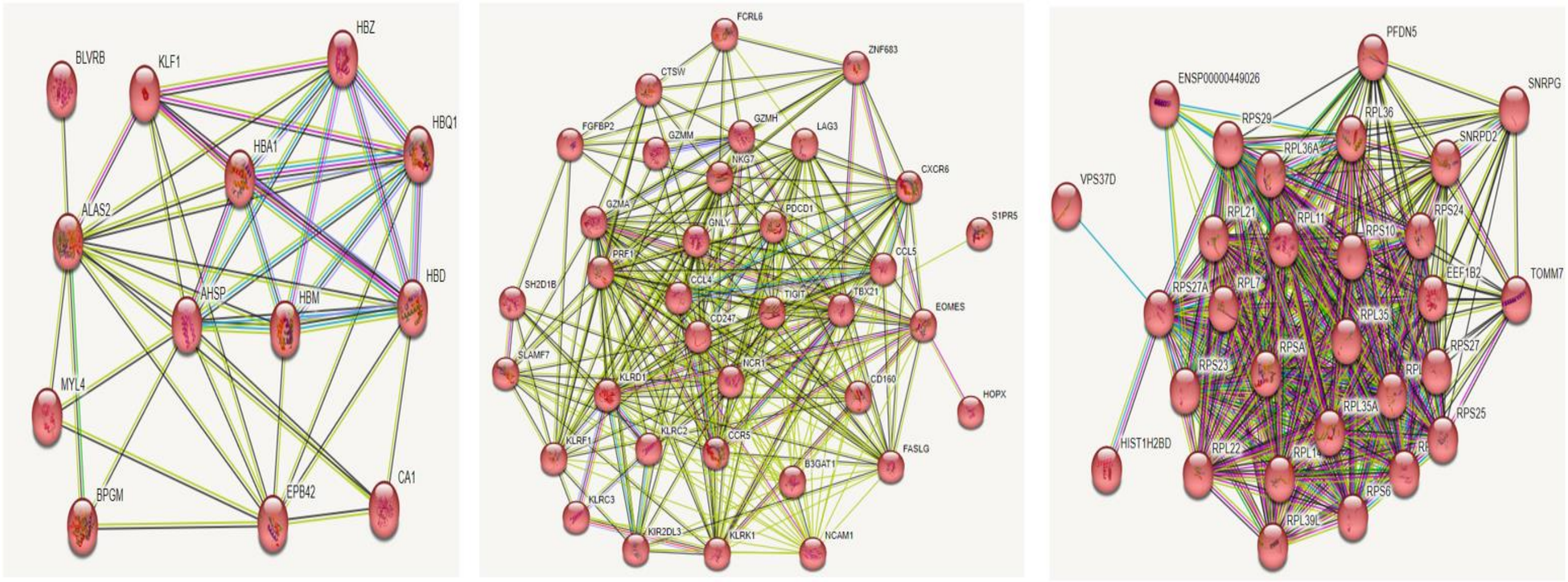

FIGURE S5 Gene network analysis of modules in KT related to R or NR. (a) The visualization of the gene network in the black modules that are positively correlated with R. (b) The visualization of the gene network in the midnightblue and grey modules that are positively correlated with NR.
